# Recent investigations of ergot alkaloids incorporated into plant and/or animal systems

**DOI:** 10.3389/fchem.2015.00023

**Published:** 2015-03-25

**Authors:** James L. Klotz, Darrin L. Smith

**Affiliations:** ^1^Forage-Animal Production Research Unit, United States Department of Agriculture-Agricultural Research ServiceLexington, KY, USA; ^2^Department of Chemistry, Eastern Kentucky UniversityRichmond, KY, USA

**Keywords:** ergot alkaloids, livestock, animal systems, plant systems, fungus

Ergot alkaloids have been associated with endophyte-infected grasses (e.g., the *Epichloë*, Bacon et al., 1977 and *Balansia*, Porter et al., 1979 spp.) with examples including tall fescue and fescue toxicosis in the United States (Yates et al., 1985) as well as perennial ryegrass in New Zealand (Rowan and Shaw, 1987) and Ireland (Canty et al., 2014). In addition to animals grazing these grasses being affected by alkaloid toxicities, these regions also provide hay for parts of the world where sufficient feedstuff cannot be grown to support existing livestock. The result has been increased occurrences of ergot alkaloid issues arising in areas not typically associated with pasture-based agriculture. To illustrate, weight loss in camels in the United Arab Emirates consuming an imported ergovaline-containing endophyte-infected perennial ryegrass straw (Alabdouli et al., 2014) along with issues associated with import and feeding of perennial ryegrass straw to Japanese black cattle (Miyazaki et al., 2001) have been documented. In addition to these incidents, grasses can also become infested with *Claviceps purpurea* where the alkaloids, typically ergotamine and ergocristine, are responsible for the resultant ergotism associated with *C. purpurea*. The presence of these toxins can compound livestock issues with the concomitant consumption of ergovaline produced by the endophyte. In terms of livestock production systems, associated ergot alkaloid toxicities are not limited to pasture or feeding pasture products. While *Claviceps Africana* is widespread throughout Africa and Asia, the first reported case of toxicity was in Australian sorghum in 1996 (Ryley et al., 1996). The *C. Africana*-infested sorghum has been demonstrated to be detrimental to steer performance in Australian feedlots that utilize this feedstuff (Blaney et al., 2011) and is an example of how ergot-contaminated feed can distress intensive livestock production.

While ergot alkaloid incidences are rare in humans resulting from increased regulation of grain processing (Flieger et al., 1997; EFSA, 2012), reports are still present from occasional pharmacological overdose or accidental exposure (e.g., Stange et al., 1998). More broad aspects of alkaloid-derived problems still persist in intensive and extensive livestock systems. The impact of ergot alkaloids has a global footprint and a large economic influence on agricultural industries. While difficult to place an exact dollar amount on the global cost from ergot alkaloids, several estimates regarding the cost of ergot alkaloids (as fescue toxicosis) have been projected in the southern United States. Hoveland (1993) estimated over $600 million in annual beef cattle losses from reduced calf births and lower weaning weights. Strickland et al. (2011) expanded this estimate to exceed $1 billion annually with the inclusion of the negative impact to small ruminant and equine industries. The human population is estimated to climb and stabilize at ~9 billion by 2050 (Lutz and Samir, 2010). As prices and global demand for meat and other animal products continue to rise, concentration of livestock production systems will also rise. If unchecked, financial losses and vulnerability of the food supply to toxins (including ergot alkaloids) will also increase proportionally (Bryden, 2012).

If fungi that synthesize ergot alkaloids pre-date the human race, and knowledge of ergot properties has been recorded as far back as 1100 BC (Schiff, 2006), why have associated problems with ergot alkaloid consumption not been solved? The primary aspect limiting progress can be attributed to the number of interactions associated with alkaloid production. The plant and fungus (endophytic or parasitic) have an interaction that is still being defined. The plant-alkaloid symbiont interacts with the ambient environment and environmental influences can impact alkaloid production. In addition to plant–fungus–environment interaction variations, the grazing animal will also influence alkaloid production. Consumption of ergot alkaloid-containing feedstuff will interact with the gut microbiome prior to the animal and likely influences the level of exposure to ergot alkaloids by the animal (De Lorme et al., 2007; Ayers et al., 2009). Biological activity of ergot alkaloids absorbed by the animal is defined by the structural similarity of these compounds to biogenic amines (Berde, 1980) allowing ergot alkaloids to interact with serotonergic, adrenergic, and dopaminergic receptors that exist in varying populations throughout the body and results in variable negative effects. In addition, limited progress can be attributed to the availability of standard reference materials or validated methods/tools to accurately extract and measure ergot alkaloids from biologic matrices. In the case of ergovaline, analytical standards for this compound are not readily available; therefore, this compound must be custom synthesized. If pure standards are not available, then actual quantities cannot be obtained and only relative responses between data sets can be generated. If pure standards can be acquired, then validation of extraction and analytical methods (using specific equipment and/or chemical instrumentation) for ergot alkaloids found in different biological matrices must be performed to ensure results are reliable and reproducible while any potential matrix effects are minimized (Smith et al., 2009).

A multi-disciplinary approach will be needed to solve most ergot alkaloid related issues. This research topic, Recent Investigations of Ergot Alkaloids Incorporated into Plant and/or Animal Systems, epitomizes that reality through diverse scientific approaches addressing the core issue of ergot alkaloids in agriculture. Innovative research articles highlight the numerous effects that ergot alkaloids can have on livestock (Aiken and Flythe, 2014; Duckett et al., 2014; Egert et al., 2014; Eisemann et al., 2014), improved characterizations of fungal endophytes (Young et al., 2014), clarification of the alkaloid variation within the plant (Mace et al., 2014), and how fungal infestations and subsequent alkaloid concentrations interact with the environment (McCulley et al., 2014). Furthermore, challenges such as alkaloid stability in collected samples (Lea et al., 2014), the generation of a large alkaloid source in the absence of a consistent supply for animal studies (Ji et al., 2014), a perspective on interpreting alkaloid concentrations and level of animal response (Craig et al., 2015), and rapid screening of livestock are addressed (Rosenkrans and Ezell, 2015). This collection of articles highlights both the complexity of the problem and the diverse approaches necessary to address these issues with the hope that future interest will be cultivated to solve global ergot alkaloid challenges.

## Conflict of interest statement

The authors declare that the research was conducted in the absence of any commercial or financial relationships that could be construed as a potential conflict of interest.
